# Function Decoupling and Modular Platform: Emerging Design Principles for MOF Luminescent Sensing

**DOI:** 10.1021/acs.accounts.6c00178

**Published:** 2026-04-08

**Authors:** Zongsu Han, Jiatong Huo, Hong-Cai Zhou

**Affiliations:** Department of Chemistry, 14736 Texas A&M University, College Station, Texas 77843, United States

## Abstract

Alongside societal development, large-scale urgent public health crises, routine food safety concerns and persistent environmental pollution have emerged as increasingly prominent challenges, prompting growing demands for rapid and reliable chemical and biological detection. Among various sensing technologies, luminescent sensing has attracted considerable attention due to its instant response, operational simplicity, and easily visualized readouts. In this context, metal–organic frameworks (MOFs) have attracted considerable interest as heterogeneous luminescent sensing materials owing to their inherent porosity, highly structural designability, and tunable photophysical properties.

Based on these advantages, numerous MOF-based luminescent sensing materials have been developed recently. However, most of these rely on highly coupled multifunctional designs, in which all functional sites are incorporated within a single framework component. Such coupled architectures introduce significant complexity in ligand and framework synthesis, obscure mechanistic insight, and require bespoke material development along with extensive screening processes. These limitations restrict scalable fabrication, constrain rational design, and impede the translation in practical applications.

To address the challenges associated with tightly coupled multifunctional MOF components, the concept of “function decoupling” was introduced as a pathway for the rational design and construction of sensing materials. In this approach, luminescence and recognition sites are independently introduced, optimized, and assembled within the framework. Such function decoupling into discrete components simplifies synthesis, reduces redundant trial-and-error optimization, and enhances design modularity, enabling the establishment of clear structure–function relationships.

Furthermore, beyond the initial concept of function decoupling, a “modular platform” strategy is further developed, in which the decoupled functional centers are packaged as interchangeable modules and incorporated into pre-engineered MOF scaffolds with reserved insertion sites. These modules can encode spectral and energy-level matching, coordination bonding, or supramolecular interactions, allowing the platform to be programmably customized for analytes with diverse structures and properties. By selectively inserting and matching the appropriate functional modules, this approach redefines MOFs from single-purpose sensing materials into adaptable, programmable platforms, enabling broader analytical applicability, while substantially reducing the synthetic complexity, matching effort, cost, and development time.

In summary, this Account traces the evolution from traditional, highly coupled MOF architectures to function decoupled centers, and further to modular platforms. First, the function decoupling strategy, in which the luminescence and recognition centers are sequentially introduced, reduces the synthetic complexity and extensive screening and matching challenges associated with conventional designs. Based on this, decoupled interchangeable functional modules are further packaged and incorporated into pre-engineered MOFs, enabling platform-based, customizable sensing, and providing a generalizable methodology for designing practical luminescent sensing materials. Collectively, this strategy establishes a rational and scalable design paradigm that bridges fundamental structure–function understanding with practical deployment and is expected to accelerate the development of next-generation programmable sensing systems across diverse analytical and real-world applications.

## Graphical abstract

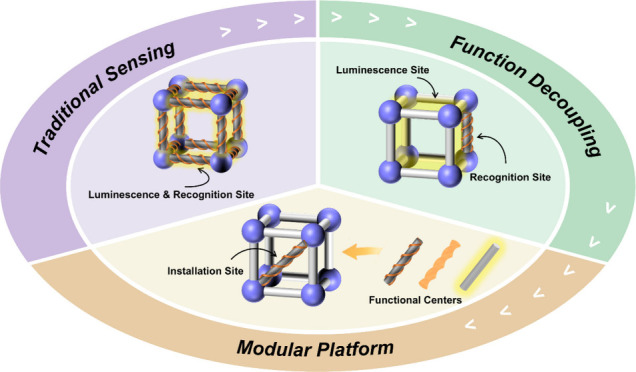

## Key References

-

Han, Z. ; Wang, K. ; Guo, Y. ; Siligardi, G. ; Yang, S. ; Chen, W. ; Zhou, Z. ; Zhang, J. ; Sun, P. ; Zhang, X. ; Shi, W. ; Cheng, P. Cation-induced chirality in a bifunctional metal organic framework for quantitative enantioselective recognition. Nat. Commun. 2019, 10, 5117 31712651 10.1038/s41467-019-13090-9PMC6848213.^1^ The representative sample for the chirality and luminescence function decoupling, which can be used for the enantioselective recognition.
-

Han, Z. ; Wang, K.-Y. ; Liang, R.-R. ; Yang, Y. ; Huo, J. ; Zhou, H.-C. Linker installation in a metal-organic framework for enhanced quantitative redox species recognition. Angew. Chem., Int. Ed. 2025, 64, e202420882 10.1002/anie.20242088239688952.^2^ The representative sample for the redox activity and luminescence function decoupling, which can be used for the redox recognition.
-

Han, Z. ; Wang, K.-Y. ; Huo, J. ; Cui, W. ; Liu, Z. ; Yang, Y. ; Liang, R.-R. ; Shi, W. ; Zhou, H.-C. Pore-engineered luminescent MOF sensors for PFAS recognition in water. J. Am. Chem. Soc. 2026, 148, 3697–3702 41531190 10.1021/jacs.5c20085PMC12856896.^3^ The representative sample for the binding site and luminescence function decoupling, which can be used for the PFAS recognition.
-

Han, Z. ; Wang, K.-Y. ; Liang, R.-R. ; Guo, Y. ; Yang, Y. ; Wang, M. ; Mao, Y. ; Huo, J. ; Shi, W. ; Zhou, H.-C. Modular construction of multivariate metal-organic frameworks for luminescent sensing. J. Am. Chem. Soc. 2025, 147, 3866–3873 39810294 10.1021/jacs.4c17248PMC11783584.^4^ The representative sample for the modular luminescent sensing platform, which can be used for the recognition toward various targets.

## 1 Introduction

Modern society faces the persistent convergence of environmental contamination, food safety risks, and unforeseen public health emergencies.^5–7^ From chronic exposure to industrial pollutants to sudden outbreaks of harmful biological agents, these challenges highlight a critical analytical need in which detection technologies must be rapid, reliable, and deployable outside specialized laboratory settings. In practical scenarios, the effectiveness of a sensing system depends not only on its sensitivity and selectivity but also on the speed and flexibility with which it can be developed and implemented.

Luminescent sensing offers distinct advantages in addressing these needs, as it relies on signals that can be generated and recorded in real time without damaging the sample.^8–10^ The simplicity of optical instrumentation, together with high signal-to-noise ratios and tunable emission characteristics, makes luminescence particularly attractive for rapid screening and long-term monitoring.^11–13^ Within this context, metal–organic frameworks (MOFs) serve as a highly versatile platform, in which ordered porous architectures enable precise spatial confinement of guest molecules, while reticular structures allow controlled incorporation of functional building blocks.^14–18^ Notably, MOF-based luminescent sensing offers distinct advantages in heterogeneous detection, particularly in terms of structural robustness and ease of separation, which enable convenient recovery and reuse of the sensing materials. Moreover, the photophysical properties of MOFs can be finely tuned through linker design, metal-node regulation, and guest incorporation, providing multiple strategies to modulate the emission intensity, wavelength, and energy-transfer pathways.^19–21^ Consequently, MOFs have been extensively explored as heterogeneous luminescent materials for the sensing of cations and anions, volatile organic compounds, persistent organic pollutants, and biological molecules.^22–28^

However, prevailing strategies for constructing MOF-based luminescent sensing materials remain heavily reliant on integrative multifunctional designs. In most cases, recognition motifs and emissive components are either combined within a single multifunctional ligand or incorporated simultaneously through one-step MOF assembly (Figure 1).^29,30^ Although the design appears straightforward, such architectures impose significant synthetic burdens. The preparation of multifunctional ligands that integrate targeting response groups with luminescent units often involves lengthy synthetic sequences, demanding a high level of synthetic expertise and precise structural control. Even after successful framework assembly, achieving satisfactory sensing performance typically requires extensive optimization, screening, and matching processes. Moreover, this intrinsic function interdependence complicates mechanistic interpretation, as recognition and signal transduction processes are hard to be examined independently.

**Figure 1:**
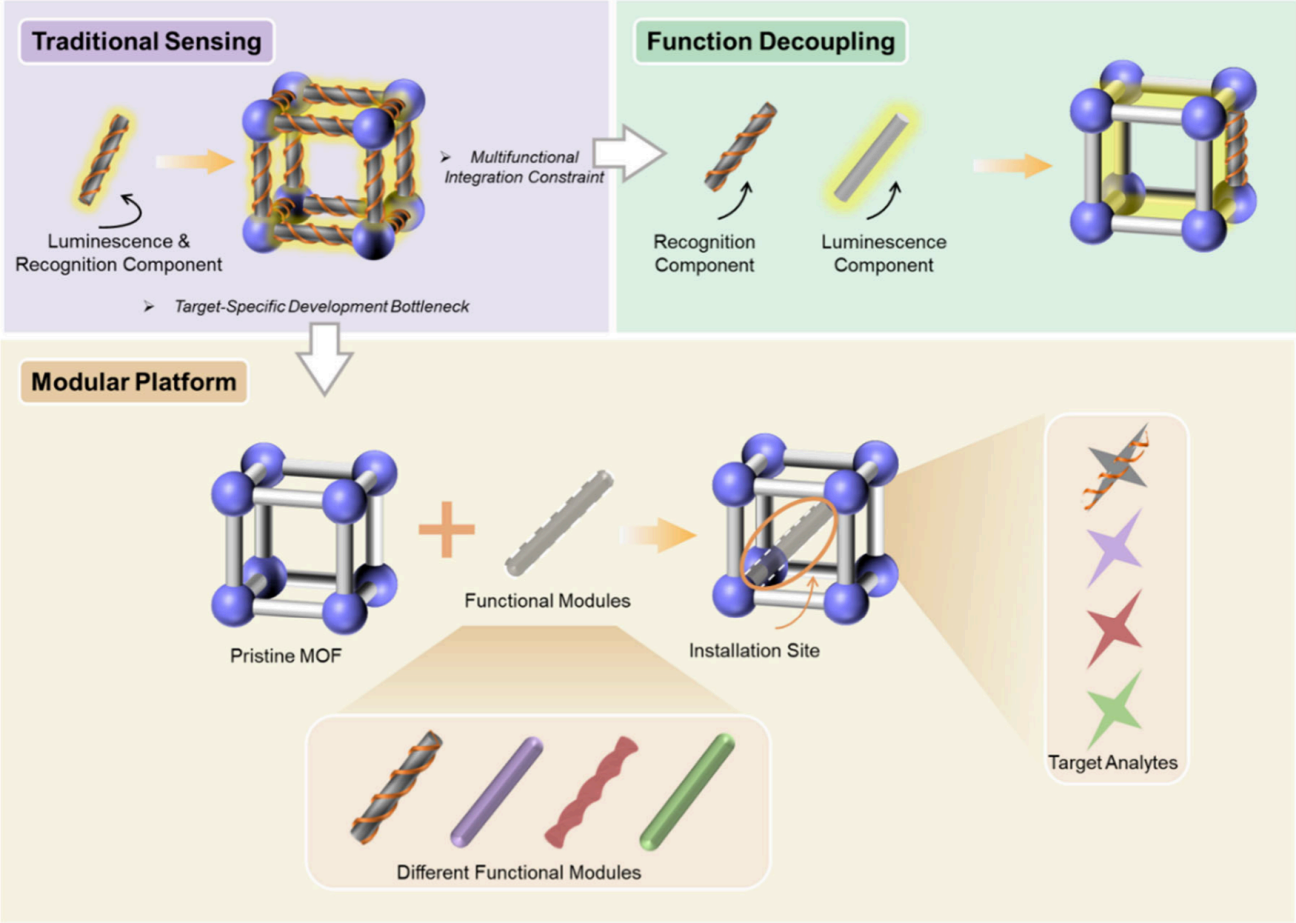
Comparisons of the construction of MOF-based luminescent sensing materials through traditional pathway, function decoupling strategy, and modular platform method.

Equally limiting is the prevalent target-specific development model. Many reported luminescent MOFs are tailored for individual analytes, requiring new ligand synthesis and framework construction for each sensing task.^29,30^ While effective for proof-of-concept demonstrations, this approach is inherently inefficient and costly. In practice, it becomes particularly impractical when rapid material development is required in response to newly identified analytes or urgent analytical demands. Therefore, the lack of a transferable design strategy represents a critical bottleneck in translating MOF-based luminescent sensing from laboratory research to practical applications.

Motivated by these challenges, the need to reconceptualize the design strategy for constructing sensing functionality within MOF systems was recognized. Rather than embedding all required roles into a single structural component, separating recognition-response sites, such as potential coordination sites, donor/acceptor motifs, reactive functional groups, and luminescent function sites, including emissive metal centers, linkers, guests, into distinct, independently tunable elements was explored. This conceptual shift, referred to as function decoupling (Figure 1), enables independent optimization and targeted incorporation of each component while replacing the need for conventional single-component multifunctional designs.^1–3^ Extending this idea further, a modular platform-based architecture was constructed (Figure 1), in which functional elements are treated as interchangeable modules that can be incorporated into predesigned MOF scaffolds according to analytical requirements.^4^ This strategy moves beyond analyte-by-analyte material discovery toward a programmable system capable of addressing diverse sensing challenges without reconstructing the entire framework. Through this approach, MOFs evolve from isolated sensing materials into adaptable infrastructures for efficient luminescent sensing.

## 2 Function Decoupling

Conventional MOF-based luminescent sensing materials are effective for detecting structurally and compositionally uncomplicated analytes, such as metal ions or simple organic molecules.^29,30^ In these cases, satisfactory recognition performance can often be achieved with manageable synthetic complexity by incorporating appropriate response motifs into the ligand backbone. The structural requirements for target binding and signal generation are largely compatible, enabling integrative ligand design without substantial compromise.

However, this paradigm becomes increasingly strained when applied to structurally complex molecules, closely related isomers, or chemically reactive species. Representative examples include enantiomers, constitutional isomers, and redox-active analytes, for which subtle stereochemical or electronic differences should be translated into discernible optical outputs. In such cases, incorporation of reactive sites directly into the ligand may lead to undesired reactions during MOF synthesis, resulting in loss of function. Consequently, both ligand synthesis and framework construction become disproportionately complicated.

Enantiomer recognition is a particularly illustrative example. Traditional strategies typically rely on the synthesis of intrinsically chiral luminescent ligands, which are subsequently assembled into chiral emissive MOFs. Achieving enantioselectivity requires low-symmetry ligand architectures to generate chiral environments, whereas efficient luminescence generally favors rigid and highly conjugated systems. These structural requirements are not inherently compatible. As a result, researchers often resort to structurally elaborate motifs such as 1,1′-bi-2-naphthol (BINOL)-derived frameworks (Figure 2a) or Schiff-base-type chiral units,^22,31^ which require highly advanced synthetic skills and involve complex synthetic procedures. Moreover, synthetic difficulty further increases when such bulky and low-symmetry ligands are incorporated during MOF crystallization. Crystallization under reduced symmetry is intrinsically less favorable, and the incorporation of extended π-conjugated ligands can further complicate framework assembly. Interpenetration frequently occurs due to the tendency of frameworks to form additional stabilizing interactions, which enhances overall structural stability (Figure 2b),^32^ leading to diminished porosity, thereby undermining the accessibility essential for sensing.

**Figure 2:**
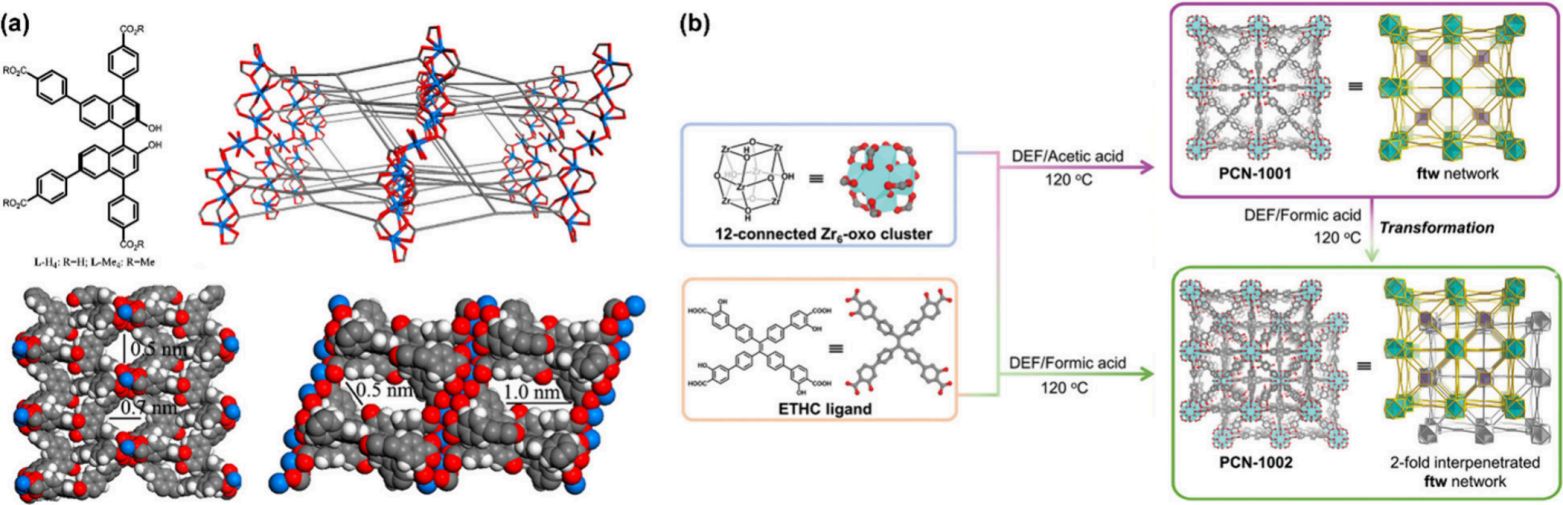
(a) The structure of an example of a BINOL-derived ligand and the chiral luminescent MOF constructed from it. Reproduced with permission from ref 22. Copyright 2012, American Chemical Society. (b) The structure of an example of a highly conjugated ligand and its noninterpenetrated and 2-fold interpenetrated MOFs. Reproduced from ref 32. Copyright 2024, The Authors.

Collectively, these challenges originate from a fundamental architectural constraint: recognition and luminescence are tightly integrated within a single structural component. When multiple demanding functions coexist in a single molecular scaffold, synthetic complexity escalates, structural predictability declines, and mechanistic interpretation becomes obscured.

To address these inherent challenges, the intrinsic structural tunability and functional modularity of MOFs was leveraged. Rather than embedding chirality and luminescence within a single multifunctional ligand, a function decoupling strategy was proposed in which recognition and emissive elements are separated. A robust luminescent framework serves as the signal-generating platform, while chiral or other target-specific units are introduced stepwise through targeted modification strategies. By disentangling stereochemical encoding from photophysical generation, each functional element can be independently optimized without imposing mutually conflicting structural constraints. This decoupled architecture not only alleviates synthetic burden but also increases the design flexibility.

Specifically, a variety of targeted modification strategies can be employed to achieve function decoupling. For example, through guest ion exchange, ionic chiral centers and emissive sites can be sequentially introduced into ionic luminescent frameworks, enabling the construction of chiral multiemissive MOFs for enantioselective sensing (Figure 3a).^1^ A luminescent ionic Zn-MOF was modified with *N*-benzylquininium and Tb^3+^ ions through this method to construct Zn-MOF–C-Tb with chiral and dual luminescent centers. Similarly, chiral coordination modification allows the introduction of chiral molecules onto luminescent MOFs via coordination bonds, which are significantly stronger than electrostatic interactions used in guest ion exchange, thereby enhancing the stability of the system (Figure 3b).^33,34^ In this manner, IRMOF-74-I/II were postcoordinated with L-lactic acid and Tb^3+^ ions to construct chiral multiemissive I/II–C–Tb. As a special case, chiral linker installation, a variant of coordination modification, leverages multiple coordination interactions to further improve the material robustness (Figure 3c).^35^ PCN-700 was employed as the prototype framework and installed with D-camphorate to form PCN-700-C. In addition, chiral defect engineering introduces chiral defect modulators to create larger pore spaces, expanding the accessible range of analytes (Figure 3d).^36,37^ UiO-66, MIL-125-NH_2_, and MIL-53 were used to validate the effectiveness of this approach in constructing chiral defective luminescent MOFs. Beyond decoupling chirality from luminescence, other binding or matching function sites can also be introduced into the framework through targeted modification strategies, allowing these strategies to regulate MOF photophysical properties,^38^ tune energy levels,^39^ incorporate specific functional groups (Figure 3e),^3^ or introduce targeted active sites (Figure 3f),^2^ providing a versatile toolbox for the rational design of target-specific luminescent sensing materials. Linker installation was further applied to PCN-700 to construct the target frameworks, thereby confirming this approach.

**Figure 3:**
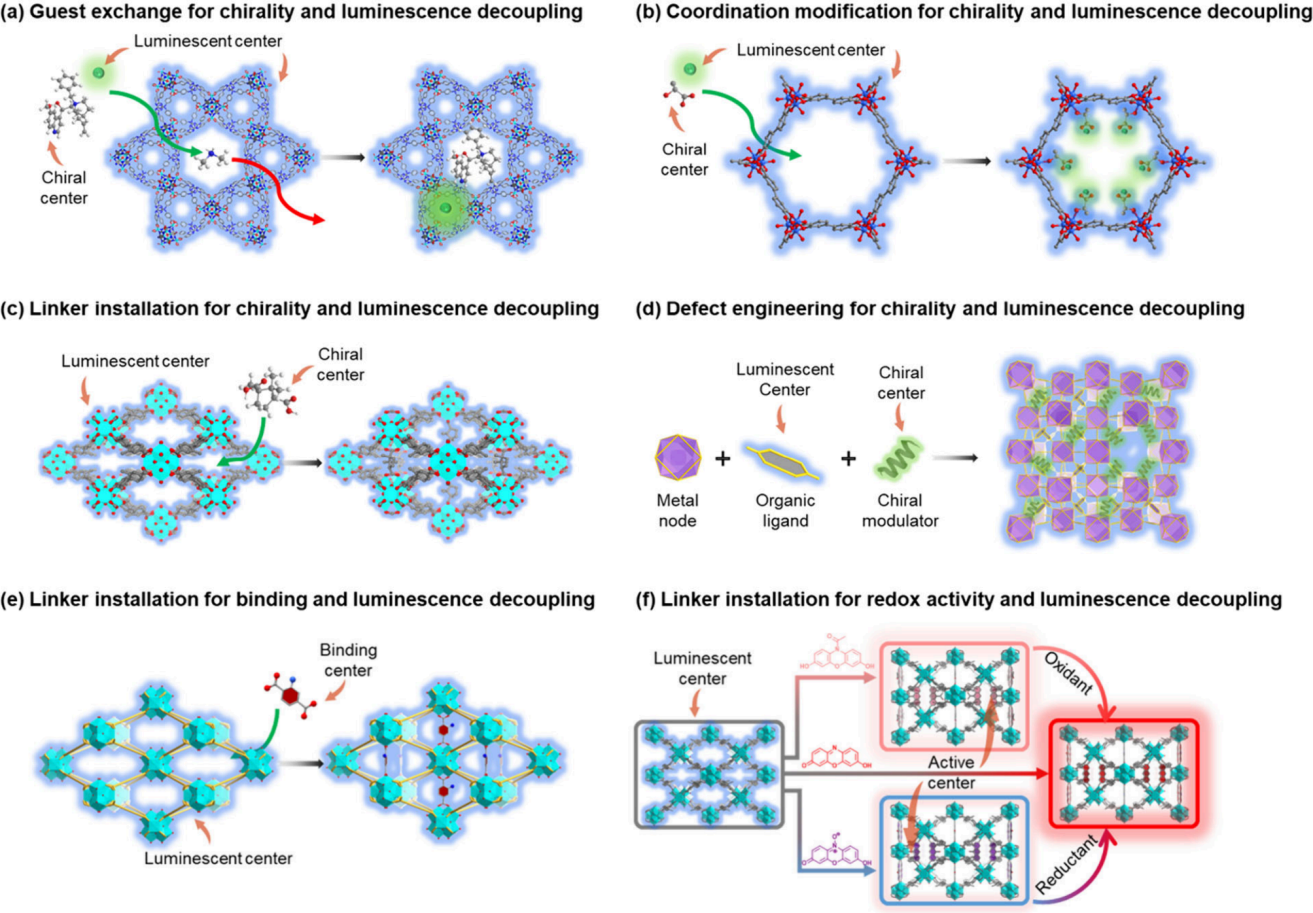
(a) An example of guest exchange method for chirality and luminescence decoupling. Reproduced from ref 1. Copyright 2019, The Authors. (b) An example of coordination modification strategy for chirality and luminescence decoupling. Reproduced with permission from ref 33. Copyright 2022, Wiley-VCH GmbH. (c) An example of linker installation method for chirality and luminescence decoupling. Reproduced from ref 35. Copyright 2024, The Authors. (d) An example of defect engineering pathway for chirality and luminescence decoupling. Reproduced with permission from ref 36. Copyright 2023, Elsevier Inc. (e) An example of linker installation method for binding and luminescence decoupling. Reproduced with permission from ref 3. Copyright 2026, American Chemical Society. (f) An example of linker installation method for redox activity and luminescence decoupling. Reproduced with permission from ref 2. Copyright 2024, Wiley-VCH GmbH.

## 3 Modular Platform

As discussed above, function decoupling enables the independent optimization of recognition-response sites and luminescent function sites. By selecting luminescent MOFs with distinct structural characteristics and integrating tailored recognition-response sites through various modification strategies, the sensing materials can be rationally configured for analytes with diverse structures and properties. Such an approach not only reduces synthetic complexity and associated material development costs, but also lowers the difficulty and expense of matching sensing materials with specific targets. In addition, it facilitates clearer mechanistic elucidation of the recognition and response processes.

Nevertheless, if implemented in a case-by-case manner, such decoupled design may still face substantial practical limitations. Each new analyte will require independent selection of framework scaffolds, recognition-response sites, and targeted modification strategies, leading to repeated material development and parallel production pathways. Although scientifically flexible, this model remains inefficient for real-world deployment.

To address this scalability challenge, we further advance the concept toward a modular platform (Figure 4a).^4^ Rather than redesigning sensing materials for each individual target, we propose the construction of MOFs that are pre-engineered with multiple accessible modification sites. These frameworks serve as universal luminescent backbones capable of accommodating diverse functional insertions. Recognition elements with distinct structures and properties can be preassembled as discrete functional modules. Target special analyte, the appropriate recognition-response module can be selectively introduced into predefined sites through compatible synthetic strategies based on the recognition-response mechanisms, such as competition absorption, photoelectron transfer, Förster resonance energy transfer, structural decomposition and transformation (Figure 4b).^4,19^ In this paradigm, sensing material development shifts from repetitive one-step synthesis to programmable module selection and insertion. The framework remains constant, while functionality becomes interchangeable and demand-driven. Such a modular architecture not only streamlines material production and reduces the developmental redundancy, but also enables standardized synthetic protocols, thereby facilitating scalable fabrication and accelerating practical translation.

**Figure 4:**
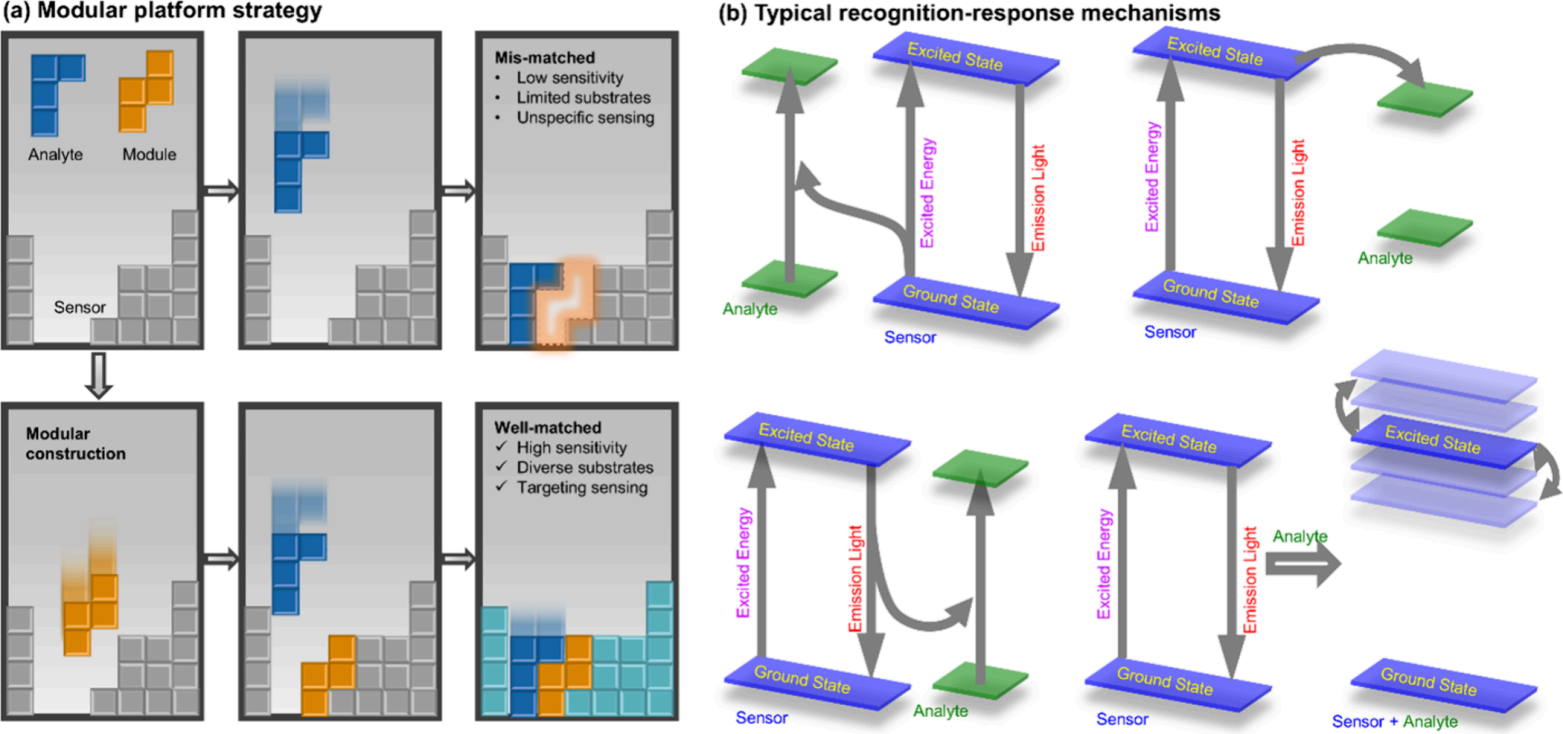
(a) Design principle for the modular luminescent sensing platform strategy. (b) Typical luminescent recognition-response mechanisms. Reproduced from ref 4. Copyright 2025, The Authors.

Building on this conceptual principle, a concrete, experimentally validated implementation of a MOF-based modular luminescent sensing platform was reported.^4^ As a representative scaffold, a structurally robust and chemically stable parent luminescent MOF with well-defined pore environments and periodically distributed anchoring sites was selected, which serves as reserved positions for functional module installation. Rather than designing special framework for each target analyte, a library of discrete recognition-response modules with tailored spectra, energy levels, and binding groups was systematically constructed by a ligand co-occupancy strategy (Figure 5). Different modules were selectively integrated into the scaffold according to the structures and properties of target analytes, including their characteristic spectroscopic/energy-level features, specific donor/acceptor sites, and reactive functional groups, through competition absorption, photoelectron transfer, Förster resonance energy transfer, structural decomposition or structural transformation mechanism-guided processes,^19,40^ while preserving the crystallinity or porosity of the parent framework. These modules enable the sensing platform to exhibit excellent sensing performance toward eight different classes of analytes. This work enables targeted recognition toward multiple distinct analytes based on single framework system efficiently for the first time.

**Figure 5:**
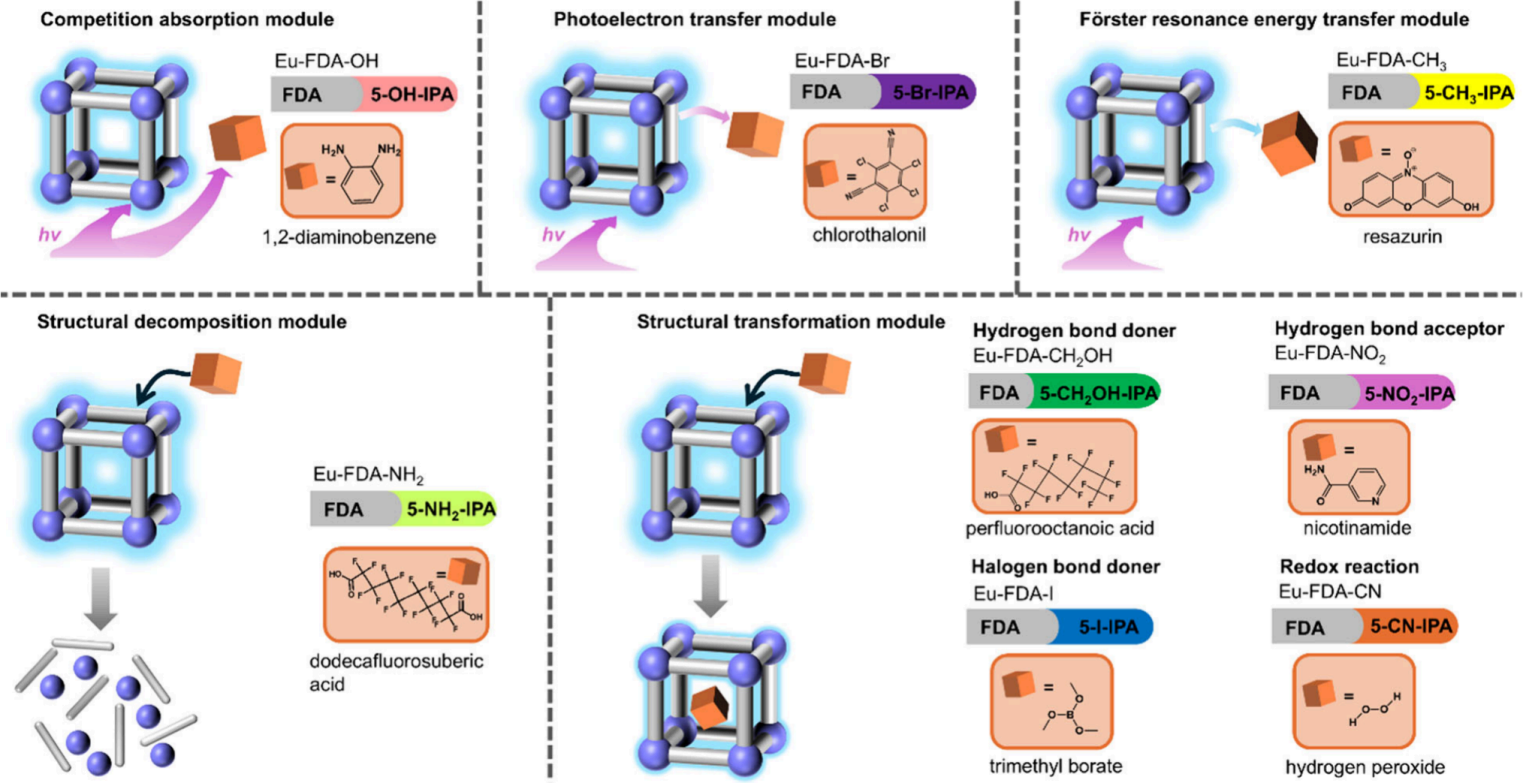
An example of the selection principle of the modules in a MOF-based modular luminescent sensing platform. Reproduced from ref 4. Copyright 2025, The Authors.

Further in our subsequent work, a variety of specialized functional modules are developed to expand the sensing capabilities of the platform, which can roughly be classified into three types according to their modes of regulation: (1) supramolecular interaction modules, featuring hydrogen-bond donors/acceptors, halogen-bond donors/acceptors, π-interaction sites, chiral binding sites, and anionic/cationic electrostatic binding sites; (2) bond-matching modules, encompassing metal/ligand coordination sites, redox/active reaction sites, substitution sites, and disruption sites; and (3) energy-level regulation modules, covering key parameter controls such as excitation/emission spectra and excited-state energy levels. These modules can serve as specialized functional units, and a corresponding module library is currently under construction to enable broader and more versatile analyte recognition.

## 4 Conclusion and Outlook

In summary, the development from “function decoupling” to “modular platforms” provides a transformative pathway for developing more effective luminescent sensing materials. By separating recognition-response sites from luminescent functional sites and integrating them in a stepwise manner, each component can be independently optimized, enabling more efficient synthesis, enhanced sensing performance, and clearer mechanistic understanding of structure–function matching. The modular design of this strategy further allows functional elements to be selectively incorporated into predesigned MOF scaffolds, thereby overcoming the limitations associated with conventional single-component multifunctional designs and analyte-specific material discovery.

Looking forward, these strategies can be further advanced by integrating diverse structural design and pore engineering approaches to construct preorganized MOF platforms with multiple addressable and readily modifiable sites. For example, MOFs incorporating different metal centers or mixed ligands as the prototype framework may offer expanded opportunities for pore engineering approaches and functional diversification. Such preconfigured scaffolds would allow more functional modules to be packaged and introduced in a programmable manner, enabling precise spatial arrangement and cooperative interactions among different components. Further, for certain analyte, multiple modules targeting different physicochemical properties may be simultaneously incorporated, allowing synergistic interactions that further amplify sensing responses and improve detection performance. By combining rational framework design with directed module insertion, it becomes possible to accommodate analytes with widely varying sizes, polarities, coordination preferences, and reactivity profiles. This strategy is expected to facilitate efficient detection of a broader spectrum of targets without reconstructing the entire framework for each new analyte. Through the development of versatile, site-defined MOF infrastructures, luminescent sensing can evolve toward a truly generalizable and adaptable platform capable of addressing increasingly complex analytical demands.
